# Metagenomic Analysis Reveals Symbiotic Relationship among Bacteria in *Microcystis*-Dominated Community

**DOI:** 10.3389/fmicb.2016.00056

**Published:** 2016-02-02

**Authors:** Meili Xie, Minglei Ren, Chen Yang, Haisi Yi, Zhe Li, Tao Li, Jindong Zhao

**Affiliations:** ^1^Key Laboratory of Algal Biology, Institute of Hydrobiology, Chinese Academy of SciencesWuhan, China; ^2^University of Chinese Academy of SciencesBeijing, China; ^3^State Key Laboratory of Systematic and Evolutionary Botany, Institute of Botany, Chinese Academy of SciencesBeijing, China; ^4^College of Life Science, Peking UniversityBeijing, China

**Keywords:** metagenome, binning, *Microcystis*, bloom, symbiosis

## Abstract

*Microcystis* bloom, a cyanobacterial mass occurrence often found in eutrophicated water bodies, is one of the most serious threats to freshwater ecosystems worldwide. In nature, *Microcystis* forms aggregates or colonies that contain heterotrophic bacteria. The *Microcystis*-bacteria colonies were persistent even when they were maintained in lab culture for a long period. The relationship between *Microcystis* and the associated bacteria was investigated by a metagenomic approach in this study. We developed a visualization-guided method of binning for genome assembly after total colony DNA sequencing. We found that the method was effective in grouping sequences and it did not require reference genome sequence. Individual genomes of the colony bacteria were obtained and they provided valuable insights into microbial community structures. Analysis of metabolic pathways based on these genomes revealed that while all heterotrophic bacteria were dependent upon *Microcystis* for carbon and energy, Vitamin B12 biosynthesis, which is required for growth by *Microcystis*, was accomplished in a cooperative fashion among the bacteria. Our analysis also suggests that individual bacteria in the colony community contributed a complete pathway for degradation of benzoate, which is inhibitory to the cyanobacterial growth, and its ecological implication for *Microcystis* bloom is discussed.

## Introduction

Cyanobacterial blooms are mass occurrence of cyanobacterial species in water bodies and are serious threats to freshwater ecosystems worldwide (Bláha et al., 2009). Due to the increased input of nutrients into lakes and rivers, eutrophication has become a global issue and it is recognized as one of the most important factors in the formation of cyanobacterial blooms in freshwater ecosystems. Among blooms of the several cyanobacterial genera, *Microcystis* blooms might be the most widely spread globally. The problems caused by the *Microcystis* blooms include the release of toxic secondary metabolites such as microcystins into the water environments that cause intoxication of humans and animals (Sivonen and Jones, 1999; Falconer and Humpage, 2006) and the formation of hypoxic zones in water bodies that lead to death of fish and other organisms. Accumulation of cyanobacteria and release of odorous materials also cause technical problems in water treatment plants and dramatically decrease the drinking water quality.

The formation of cyanobacterial blooms has been studied extensively and much progresses are made in understanding the relationships between the blooms and environmental factors (Conley et al., 2009; Davis et al., 2009; Ma et al., 2014). Recently, people have started to pay more attention to the importance of symbiotic relationship between *Microcystis* and the associated heterotrophic bacteria (Eiler and Bertilsson, 2004; Sigee, 2005; Berg et al., 2008; Dziallas and Grossart, 2011, 2012; Shen et al., 2011). It is desirable to obtain the genomes of these heterotrophic bacteria and understand their ecological roles in *Microcystis* colonies. However, because only a small portion of bacteria in nature can be cultured in the lab and methods are quite limited in isolating individual genomic DNA or RNA samples, the complex structures of microbial interactions, including *Microcystis*-bacteria associations, are not well understood. The successful application of metagnomics in studies of microbial communities, such as ocean water, natural acidophilic biofilm, permafrost, acetate-amended aquifers, activated and sludge bioreactors, has led to new insights and mechanisms for studying bacterial communities in natural environments (Tyson et al., 2004; Venter et al., 2004; Mackelprang et al., 2011; Wrighton et al., 2012; Albertsen et al., 2013). The metagenomics approach has also been applied to cyanobacterial blooms (Pope and Patel, 2008; Eiler et al., 2011; Li et al., 2011; Steffen et al., 2012; Mou et al., 2013). These works have provided very useful information on the microbial communities in *Microcystis* blooms. However, due to the lack of whole genome information from the individual members of the community, details on the metabolic pathways and the relationships among members of the community are not well understood.

In metagenomic analysis, grouping sequences from a particular genome from microbial community sequencing data is an important step referred to as binning (Kunin et al., 2008; Mande et al., 2012). The binning process can greatly reduce the complexity of metagenomics data by grouping similar sequences together followed by assembly and annotation to the individual genome bins. Therefore, it enables researchers to decipher the functional roles of community members and their interactions. Currently, there are several programs designed to classify sequencing reads (Huson et al., 2007; Wu and Ye, 2011; Wang et al., 2012) from either metagenomic samples or assembled scaffolds (Dick et al., 2009; Albertsen et al., 2013). Recently, model-based clustering techniques, in particular the multi-component Gaussian mixture model, have been employed to bin metagenomic fragments (Alneberg et al., 2014; Laczny et al., 2014; Wu et al., 2014).

Here, we present a novel visualization-guided and model-based binning method. It was applied to the metagenomic dataset generated from a *Microcystis*-dominated bloom in Lake Taihu. The high-resolution binning results show that there were at least seven bacteria species in stable cohabitation with *Microcystis* species. The functional annotation and pathway analysis provided an insight into the relationships among *Microcystis* and the associated bacteria. It suggests that metabolic pathway complementation plays an important role in stable colony formation and the long-term domination of *Microcystis* blooms. A possible impact of *Microcystis*-bacteria association on health effects for both humans and animals is discussed.

## Materials and methods

### Strains

**Figure 1:**
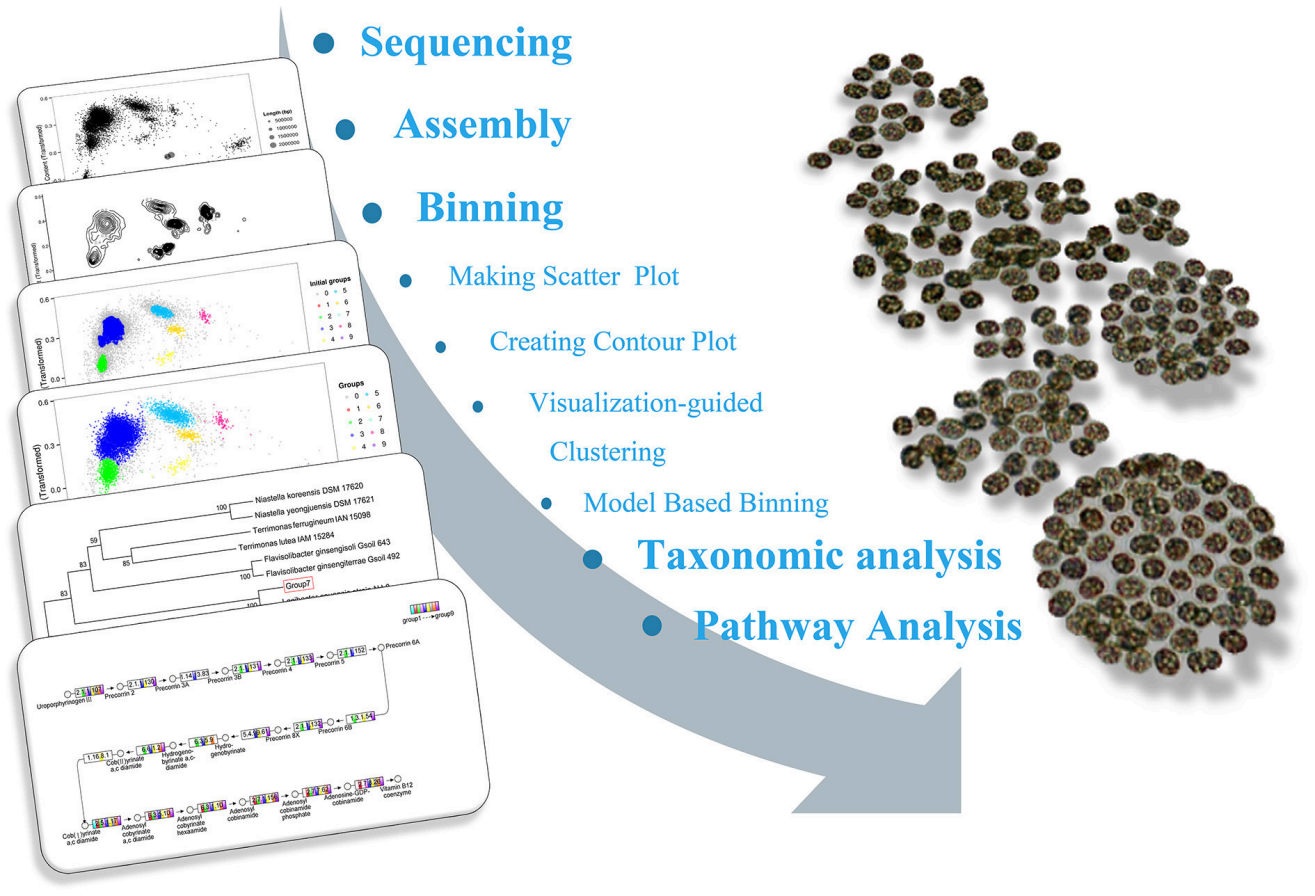
**Overview of the pipeline to bin genomes of each group from the *Microcystis*-dominated bloom metagenome**. The screenshots in the left part of the figure correspond to the steps for analysis the datasets. Following the process, the association between *Microcystis* and attached heterobacteria on the metabolic level can be elucidated effectively.

*Microcystis wesenbergii* T100 was isolated from Lake Taihu in 2006, a freshwater shallow lake in East China in which serious cyanobacterial blooms occur annually. Non-microcystin-producing *M. wesenbergii* T100 forms large mucilaginous colonies that have embedded several species of bacteria. The cultures were maintained in BG-11 medium in an incubation room at 25°C under 25 μM photons m^−2^ s^−1^ on a 12:12 light-dark cycle. A flow diagram of the binning and downstream analysis process was shown in Figure 1, and a step-by-step guide is available at http://mingleir.github.io/meta-binning/.

### Metagenomics DNA sequencing

In 2009, the cells were harvested in the exponential phase by filtering water samples through a 0.45-μm pore filter and disrupted with Mini-Beadbeater (BioSpec Products, Elgin, IL, USA) at maximum speed. Total DNA was extracted according to Li et al. (2001) with modifications. Briefly, the sample was suspended in TE buffer (pH 8.0) with 0.5% SDS and 1 mg/mL lysozyme incubated at 37°C for 1 h. Before digesting at 55°C for 2 h with 0.4 mg/mL proteinase K, 1% β-mercaptoethanol was added to prevent pigment oxidation. The mixture was purified with phenol-chloroform-isoamyl alcohol (25:24:1) and washed with 70% ethanol twice. The final supernatant was precipitated with isopropanol and dissolved in sterile water. A 300-bp paired-end library and a 3-Kb mate-paired library were constructed according to the manufacturer's instructions (www.Illumina.com). The libraries were sequenced on an Illumina Genome Analyzer IIx following the manufacturer's instructions with 2 × 81 bp read length.

### Data preprocessing and assembly

Quality trimming was performed in Trimmomatic v0.32 (Bolger et al., 2014) with reads shorter than 20 bp being discarded. After filtering, we used SPAdes v3.0.0 (Bankevich et al., 2012) to assemble the metagenomics data of cyanobacterial blooms in Lake Taihu with different parameters. Optional parameters were selected by considering the scaffolds *N50* statistic, the number of scaffolds, and the maximum scaffold length.

### Data visualization by scatter points

In the present study, we considered both sequence composition and relative abundance in the community in the assembled scaffold cluster analysis. GC content is the most fundamental compositional feature of a genome and has long been used in the identification of unknown DNA fragments (Karlin, 1998). The most intuitive measure of abundance is coverage. Sequences belonging to the same source population should have similar coverage (Strous et al., 2012). Recently, inspired by the rapid progress of RNA-sequencing, many statistical models have been proposed to accurately quantify transcript relative abundances (Pachter, 2011). Furthermore, the problem of estimating genome relative abundances in a community is closely related to relative transcript abundance estimation (Pachter, 2011). Fragments per kilo base per million mapped reads (FPKM) (Trapnell et al., 2010), defined as the read coverage normalized by the total number of mapped reads and sequence lengths, are widely accepted as a measure of quantifying relative transcript abundance and we used FPKM as a proxy for relative genome abundance in a community. We used Bowtie v. 1.0.1 (Langmead et al., 2009) to count the reads mapped on the scaffolds and eXpress v. 1.5.1 (Roberts and Pachter, 2013) to estimate the FPKM of each scaffold.

To show the structure in the set of sequence features, two-dimensional kernel density estimation was applied to the joint density function of GC content and FPKM. Each scaffold was weighted by its length because we investigated the distribution of both features at the nucleotide level. Because bandwidth is critical in the practical implementation of kernel density estimation (KDE) (Rosenblatt, 1956; Parzen, 1962), a two-stage direct plug-in bandwidth selector (Sheather and Jones, 1991; Wand and Jones, 1995) was used to produce a bandwidth estimate for the data set. We observed that sequence coverage contained outliers with extremely large values, which can have considerable effects on bandwidth selection. Therefore, we discarded scaffolds with coverage >0.99 quantile. Furthermore, logit transformation was applied to GC content, which was represented as proportion data, so that it follows a normal distribution. Binned kernel density estimation was applied to the transformed data (Wand, 1994; Wand and Jones, 1995). The standard bivariate normal density was used as the kernel. The estimated density function was visualized by contour plots. Guided by the graphs, the correct density function value was selected to initially bin the scaffolds.

### Model-based clustering

From the initial binning graph described above, two facts could be observed: (1) each group could be modeled by a bivariate normal distribution; (2) a large number of data points (scaffolds) were scattered among the initial groups. Based on the observations, we modeled the data with a mixture of two-dimensional Gaussian distributions

$$\begin{array}{l} P\left(x_{i}\right)=\sum_{k=1}^{G}\tau_{k}\phi_{k}\left(x_{i}|\mu_{k},\Sigma_{k}\right)\;i=1,\;2,\;\ldots ,\;N, \end{array}$$

where *G* is the number of components, τ_*k*_ is the probability that an observation belongs to the *k*th component ($\tau_{\text{k}}\geq 0;\overset{\text{G}}{\underset{\text{k}=0}{\sum }}\tau_{\text{k}}=1$), *x*_*i*_ represents the observation in the data, N is the total number of scaffolds, and

$$\begin{array}{l} \phi_{k}\left(x_{i}|\mu_{k},\Sigma_{k}\right)={\left(2\pi \right)}^{-\frac{p}{2}}{\left|\Sigma_{k}\right|}^{-\frac{1}{2}}\\ \text{exp}\left\{-\frac{1}{2}{\left(x_{i}-\mu_{k}\right)}^{\text{T}}\Sigma_{k}^{-1}\left(x_{i}-\mu_{k}\right)\right\}. \end{array}$$

The number of *G* components was set to the number of initial groups plus one, which was added to accommodate data points scattered among groups. The EM algorithm starting with M-step implemented in *mclust* package (Fraley and Raftery, 2002; Fraley et al., 2012) in R (Team, 2008) was applied to fit the mixed model. Here, each scaffold was weighted by length, thus the complete data log-likelihood is

$$\begin{array}{l} l_{c}=\sum_{i=1}^{n}\sum_{k=1}^{G}z_{ik}w_{i}\text{log}\left[\tau_{k}\phi_{k}\left(x_{i}|\mu_{k},\Sigma_{k}\right)\right], \end{array}$$

where *n* is the number of data points, *z*_*ik*_ is the conditional probability that observation *i* belongs to group *k*, *w*_*i*_ is the weight of observation *i*, in this case ${\text{w}}_{\text{i}}={\text{l}}_{\text{i}}∕{\overset{\text{n}}{\underset{\text{i}=1}{\sum }}\text{l}}_{\text{i}}$, and *l*_*i*_ is the length of scaffold *i*. The algorithm requires initial estimates of conditional probabilities. To simplify, we set the *z*_*ik*_ = 1, if observation *i* belonged to group *k* in the initial binning and to 0 if it did not.

### Population genome assembly validation

Owing to the limited reference genome sequences, it is still hard to investigate the completeness of each group from metagenomics data. Generally, different types of conserved genes have been chosen to validate the integrity of the genomic fragments, such as ribosomal genes and core genes within related organisms (Hess et al., 2011). However, ribosomal genes are not sufficient to assess the real completeness and accuracy of a metagenomics assembly, and the core genes are difficult to define. In this study, we evaluated the completeness of each group by 107 essential single-copy genes, which were conserved in 95% of all sequenced bacteria (Dupont et al., 2012). We referred to the steps described in Albertsen et al. (2013). First, we predicted the open reading frames (ORFs) of each group by Prodigal (Hyatt et al., 2012), these ORFs were then searched against a set of 107 hidden Markov models (HMMs) of essential single-copy genes by HMMER3 (Johnson et al., 2010) with the default settings.

### Taxonomic and functional classifications

To understand the composition of this community, the predicted ORFs of each group are aligned to the NCBI-NR database using BLASTP with a maximum *e*-value of 1e-5. The blast outputs were then filtered (at least 40% amino acid sequence identity and 50% length hit) and the filtered results (each line was repeated by its FPKM) were imported into MEGAN5 (Huson et al., 2011) for taxonomic classification. To be more exact, the 16S ribosomal RNA genes of each group were predicted by RNAmmer (Lagesen et al., 2007) and the 16S ribosomal RNA genes of their closest neighbors were downloaded from the NCBI, the alignments were run in MEGA5 (Tamura et al., 2011) (neighbor-joining) at the default settings with 1 000 bootstraps. The predicted ORFs of each group were annotated using the GhostKOALA service (Kanehisa et al., 2012), and then the interesting KEGG pathways were analyzed comparatively. For the KEGG classification, the complementation ratio (the number of mapped genes divided by the total number of genes involved in the respective pathways) was counted (Endo et al., 2014). COGs (Clusters of Orthologous Groups) were also used to assign function (Tatusov et al., 2000).

## Results

### Analysis of metagenomics data from *microcystis* colonies with an improved binning method

Annual massive *Microcystis* blooms have become a severe ecological problem for Lake Taihu, the second largest lake in China. There are several species of *Microcystis* in the blooms and they have different colony morphology. The species used in this was *M. wesenbergii* T100 isolated from Lake Taihu. The culture was maintained in the lab for 8 years and the colony morphology was unchanged during the period of lab culture. For metagenomics study, one paired-end (PE) library with a 300-bp insert and one mate-pair (MP) library with a long insert (3 kb) were sequenced with 81 base PE reads, producing 6.6 and 3.2 Gb of raw data, respectively. After trimming and filtering, a total of 84 million clean reads were assembled into 60 Mb of scaffolds ranging in size from 1 kb to 1.2 Mb using SPAdes.

For the metagenomic datasets obtained from *Microcystis*-bacteria colonies, we developed an improved binning method, employing EM algorithm (Dempster et al., 1977) to assign individual scaffolds to each genome. We first used two genomic features, GC content and read coverage of each scaffold, as the two coordinates to generate a two-dimensional scatter-plot after DNA sequencing and assembly. The 2-D plot separated scaffolds according to the intrinsic properties of each genome. However, since the scaffolds with similar GC content and read coverage would tend to group together in the plot, further separation was needed. The values of GC content of scaffolds from a bacterial genome would fluctuate around the GC content of the whole genome. Statistically, a long scaffold would have a GC value closer to the GC value of the genome than a short scaffold and it is more reliable to assign long scaffolds to real genomic fragments of individual species than short ones or misassembled sequences. We therefore introduced scaffold length as another parameter so that each scaffold was weighted by its length and further separation of scaffolds on the 2-D plot could be achieved. The number and position of population genome bins on the graphic can be determined based on the contour plot of the joint probability density function which is generated by kernel density estimation (KDE) (Rosenblatt, 1956; Parzen, 1962). We manually determined the value of contour level for binning so that the individual bin position can be separated in the best way from the others.

We tested this binning method first with a published simulated dataset in the article (Wu et al., 2014), which was generated from 10 species by MetaSim (Richter et al., 2008). The binning results showed that the assembly was regrouped into 10 genome bins (representing 10 genomes), and the majority of each bin was correctly assigned (Supplementary Figure S1). The performance of our binning method is comparable with MaxBin's (Wu et al., 2014), particularly for the species with high abundance.

**Figure 2:**
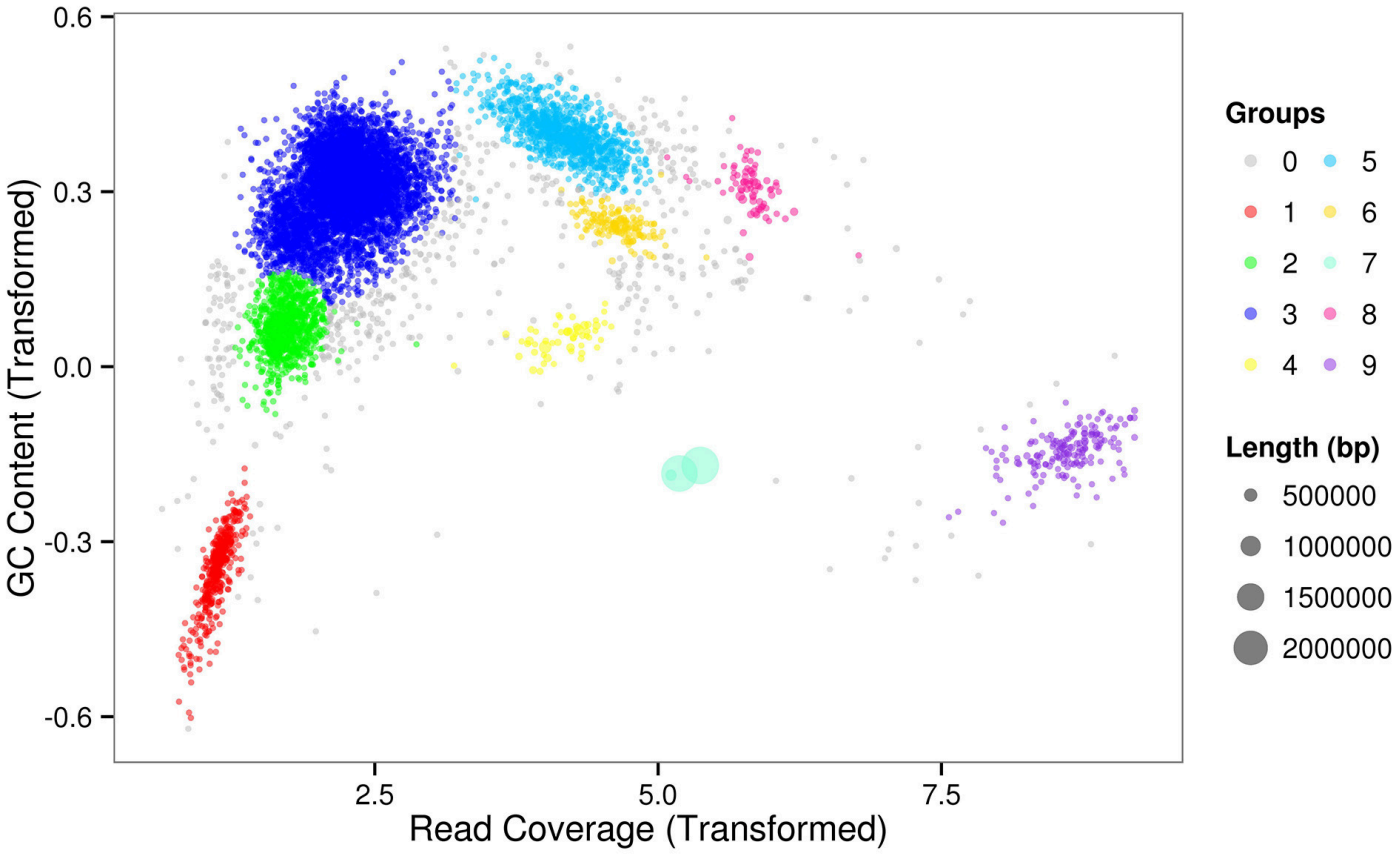
**Scatterplot of the final binning results for the metagenomics sample based on the Gaussian mixture model**. The points in the figure represent scaffolds assembled from the microbial community and the point size indicates the length of scaffolds. The colored clusters on the graph are potential genomes of each group. The gray points dispersed around the clusters are scaffolds that were difficult for the model to assign.

**Figure 3:**
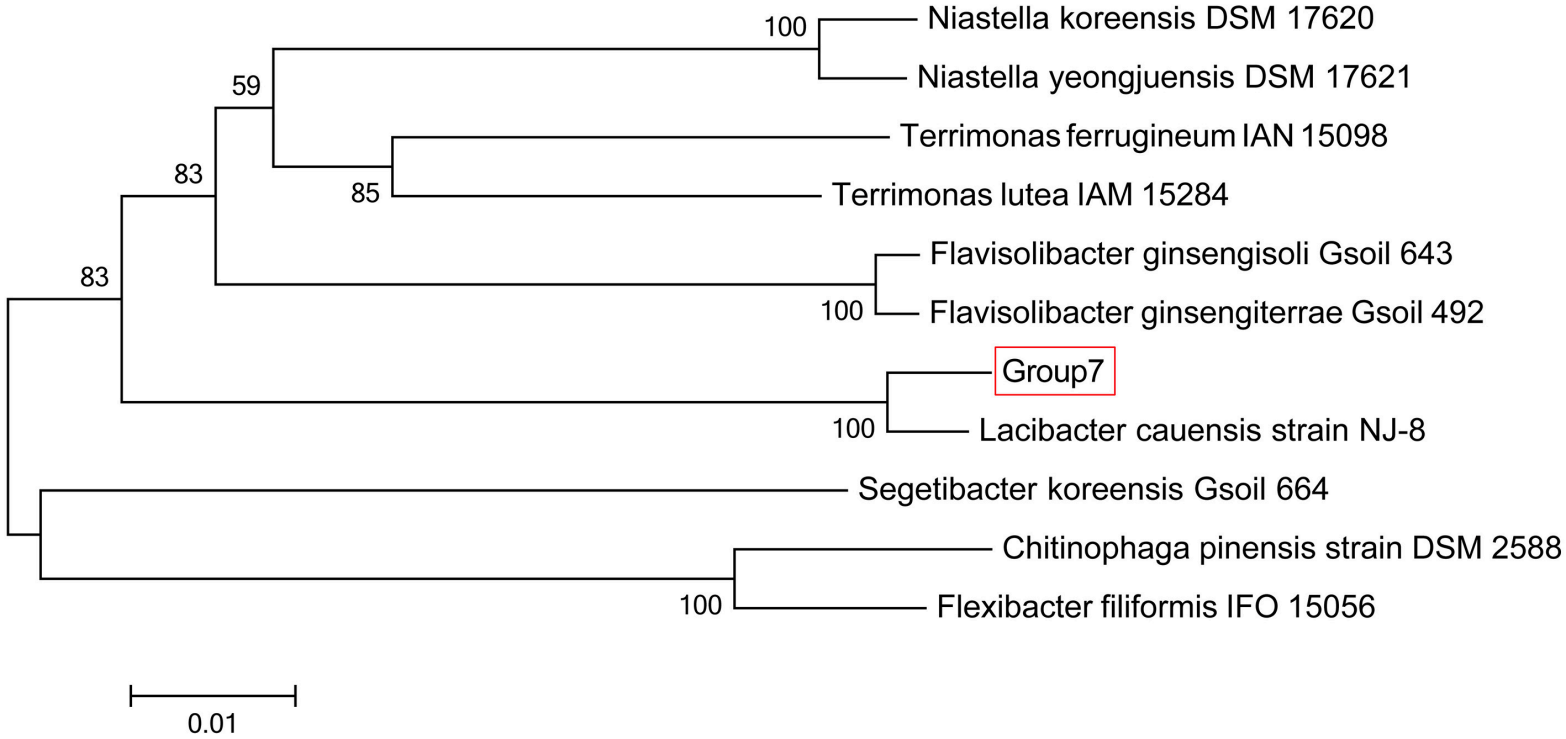
**Phylogenetic inference of Group 7 using 16S rRNA gene sequences**. Neighbor-joining phylogenetic tree is constructed based on 16S rRNA gene sequences showing the position of Group 7 among related species. Numbers at nodes indicate bootstrap percentages (based on 1000 replicates); only values >50% are shown. Bar, 0.01 substitutions per nucleotide position.

The sequencing results of *M. wesenbergii* colonies mentioned previously were analyzed with our binning method. Based on the contour-plot results (Supplementary Figure S2), we applied a Gaussian mixture model to obtain the optimal scaffold clusters. The final binning result was visualized by a scatterplot with different colors representing different population genome bins (Figure 2). In total, nine genome bins (Supplementary Table 1) were identified and the population scaffolds with a coverage of 70.88% of all clean reads. The basic information of the groups (e.g., length, GC content, and the numbers in each group) is shown in Table 1. To identify these groups taxonomically, we analyzed them by the program MEGAN5 (Supplementary Figure S3). At the phylum level, six of the nine groups were Proteobacteria, which are the most abundant in Lake Taihu (Steffen et al., 2012), two were Bacteroidetes and one was the group of Cyanobacteria. At the order level, eight of the nine groups were classified to the relevant order with maximum probability, except for Group 3, which could contain more than one order with similar GC content and abundance. At the genus level, Group 6, 7, 8, and 9 were from *Agrobacterium, Niastella, Pseudomonas*, and *Microcystis*, respectively. Group 1 and 5 were from genera that are closely related to *Flavobacterium* and *Methylobacterium*. Group 2 and 4 were both from *Limnobacter* and they had similar GC contents. Group 7 was classified to *Niastella* genus according to the result of MEGAN, but when the confirmation was performed based on the 16S ribosomal RNA, phylogenetic analysis showed that it was closer to *Lacibacter* genus (Figure 3). And one bacterium in the *Lacibacter* genus was isolated from the surface layer sediment of Lake Taihu and identified as *Lacibacter cauensis* NJ-8^T^ (Qu et al., 2009). To date, there is no reference genome for the *Lacibacter* genus, and our method provides near-complete chromosomes for this genus. This conflict between taxonomy assignments was due to the limitation of reference genomes in the analysis of metagenomics. The genomes of eight of the nine groups were estimated to be over 85% completed when compared with the number of essential genes of all sequenced bacteria at the phylum level (Dupont et al., 2012). Among the eight, five were over 95% complete (Table 1). The copy numbers of the essential genes, which indicate how well the genomes are separated from one another, were also examined and the results (Supplementary Table 2) suggested that each group except Group 3 had little contamination from other genomes. As mentioned above, Group 3 could contain more than one bacterium since the number of its essential genes was much higher. Overall, our method effectively separated groups with different GC contents and abundances, while maintaining the integrity of each group.

**Table 1:** **The basic statistics for each group of *Microcystis* colonies**.

**Group**	**No. contigs**	**Length (bp)**	**GC content**	**No. essential genes**	Relative abundance^*^(%)	**Genus**	**Phylogenetic affiliation (phylum)**
Group1	443	3,239,598	33.95	100/105(95.24)	0.5	*Flavobacterium*	Bacteroidetes
Group2	943	2,750,644	53.05	46/106(43.4)	0.5	*Limnobacter*	Betaproteobacteria
Group3	5810	10,026,678	64.26	91/105(86.67)	1.8	*Rhizobium*	Alphaproteobacteria
Group4	53	3,406,030	52.26	106/106(100)	4.4	*Limnobacter*	Betaproteobacteria
Group5	1000	4,081,424	68.5	93/105(88.57)	2.3	*Methylobacterium*	Alphaproteobacteria
Group6	150	4,587,864	61.67	104/105(99.05)	4.9	*Agrobacterium*	Alphaproteobacteria
Group7	3	4,737,723	41.25	105/105(100)	14.8	*Lacibacter*	Bacteroidetes
Group8	73	4,214,643	64.34	105/105(100)	6.4	*Pseudomonas*	Gammaproteobacteria
Group9	163	4,195,911	43.06	95/106(89.62)	31.6	*Microcystis*	Cyanobacteria

* *Relative abundance was calculated as the percentage of reads of a genome bin in the total number of reads*.

### Functional analysis of the *microcystis*-dominated community

To understand the biological functions of the microbial community, genes from all groups were assigned to broad functional categories (COGs) (Supplementary Figure S4). With the exception of general function prediction only (R) and function unknown (S), most of the genes were involved in amino acid transport and metabolism (E), cell wall/membrane/envelope biogenesis (M) and translation, ribosomal structure and biogenesis (J), inorganic ion transport and metabolism (P), and energy production and conversion (C). The result shows that the quantity of each category in the Group 3 was three to four times more than that from other groups, suggesting this group contained more than one bacterium. This suggestion is supported by the result of taxonomic assignment (Table 1).

**Figure 4:**
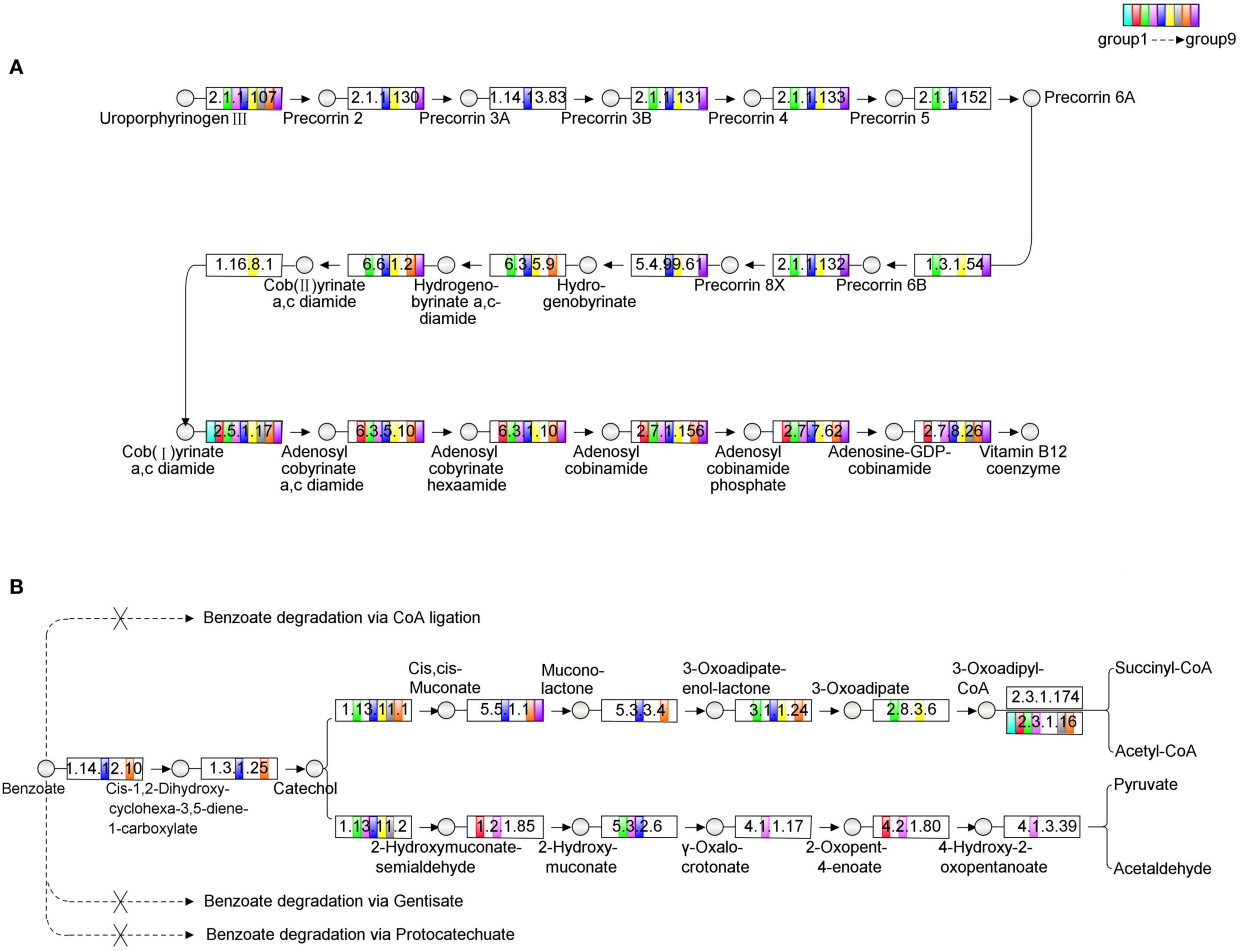
**The analysis of metabolic pathways among the microbial community**. The circles in the figure represent the intermediates, the rectangles represent the enzymes. Different filled colors of rectangles represent the groups from the community, among which the white color indicates the absence of the corresponding enzyme in this group. **(A)** The aerobic biosynthesis pathway of Vitamin B12. The enzymes tagged by red rectangles are absent in Group 5, while the enzymes tagged by blue rectangles are absent in Group 6, but the pathway is completed with the combination of Group 5 and 6. **(B)** The metabolic pathway of benzoate degradation. In the metabolic diagram, only Group 5 and 8 participate consistently in the process of converting benzoate to 3-oxoadipate, while Group 3 and 6 then catalyze it to 3-oxoadipul-CoA with EC 2.8.3.6, which is absent in both Group 5 and 8 in our analysis. Through this kind of cooperation, the whole community can adapt for the complex environment.

*Microcystis* is the only photoautotrophic bacterium and the other bacteria are all heterotrophic in the *Microcystis*-bacteria colonies (Cole, 1982). Since the culture was maintained with a minimal medium, these heterotrophic bacteria depended on both carbon and energy source from *Microcystis*. On the other hand, analysis of *Microcystis* genomes showed that it had a methionine biosynthesis pathway that has a type-II MetH enzyme which needs vitamin B12 as cofactor (Croft et al., 2005; Kaneko et al., 2008; Helliwell et al., 2011). During the subculture, no vitamin B12 was added to culture. The possible explanation of the fact was that these heterotrophic bacteria are the likely candidates that providing vitamin B12 for *Microcystis*. An alternative was that these bacteria could provide methionine directly to *Microcystis* cells. In either case, *Microcystis* benefited from being associated with these bacteria. Therefore, the relationship between the heterotrophic bacteria and *Microcystis* is mutually beneficial. The contribution of each individual bacterium in the colonies to the biosynthesis of vitamin B12 was analyzed and the results showed that none of group could complete the aerobic biosynthesis pathway of vitamin B12 independently. The complementation ratio of the vitamin B12 biosynthesis pathway of the colonies (Supplementary Figure S5) calculated according to Endo et al. (2014) suggested that two groups, such as Group 5 and Group 6 (Figure 4A), or more of the bacteria were required for the vitamin B12 synthesis in the colonies. The complementation ratio of benzoate degradation pathway was also indicative of mutualism. Group 5 and 8 could be responsible for converting benzoate to 3-oxoadipate (Harayama et al., 1991), Group 3 and 6 then could catalyze it to 3-oxoadipul-CoA with EC 2.8.3.6, which is absent from Group 5 and 8 (Figure 4B). While the cellular mechanisms of the metabolic complementation require more studies, our analysis implied that benzoate can be degraded to acetyl-CoA and succinyl-CoA through cooperated actions of these species.

The harmful effects of *Microcystis* blooms to human and animal health are noteworthy, including the release of toxic microcystins and lipopolysaccharides (LPS). A typical LPS molecule is composed of three parts: lipid A, core polysaccharides, and O-antigen repeats. Lipid A is located in the outer leaflet of the outer membrane, while core polysaccharides and O-antigen repeats lie on the surface of the bacterial cells by attaching to lipid A. Although *Microcystis* LPS have been reported to cause some serious health issues, their synthesis are not well understood (Raziuddin et al., 1983; Snyder et al., 2009). We analyzed the LPS biosynthesis pathways (Supplementary Figure S6) and found that *Microcystis* only had genes for synthesis of lipid A and lacked the genes responsible for synthesis of other components of LPS. Figure S6 also shows that the associated bacteria had almost complete composition of LPS, suggesting that the LPS-related health problems were caused by the *Microcystis*-bacterial colonies.

## Discussion

Colony formation is one of the key features in *Microsystis* blooms and it has been shown that heterotrophic bacteria play important roles in the colony formation (Shen et al., 2011; Wang et al., 2015). The *M. wesenbergii* T100 was isolated from Lake Taihu and it maintained the ability to form colonies in subculture. The lab maintained colonies are less complex and their bacterial composition is less variable than the ones in nature and therefore they are more suitable for our initial metagenomics study of colony structures.

In analysis of metagenomic data, sequence binning is one of the most important steps for obtaining more information about composition and structure the microbial community. Comparing with single bacteria genome assembly, metagenomic assembly is more fragmental and it generates more small scaffolds. How to assign small scaffolds, which have been often treated as noise, to a respective genome is a challenge for metagenomic binning. The *N50* values (a statistic of a set of contig or scaffold lengths, widely used in genome assembly) of individual population genome bins in metagenomic assembly vary in a wide range. The scaffold number of each genomics bin varies from one to thousands, causing various problems for model-based binning. Our binning method employed the EM algorithm (Dempster et al., 1977) to assign the assembled scaffolds, which were derived from individual genomes, to their respective groups. The parameters used in our method are GC content, contig coverage and contig length. GC content is one of the most intrinsic compositional features of genomes and it is often used in the identification of unknown DNA fragments (Karlin, 1998) and contig/scaffold coverage level has been used in other binning methods (Wrighton et al., 2012; Albertsen et al., 2013). A big difference between our binning approach and other methods based on mixture model is that the joint distribution of sequence features, GC content and abundance, was dissected at nucleotide level instead of sequence level and it was accomplished by introduction of contig length as the third parameter. The finer granularity could provide higher resolution and reduce the noise of poor-assembled short scaffolds. With accurate initial values of parameters obtained by kernel density estimation (KDE) (Rosenblatt, 1956; Parzen, 1962), the number of iteration in calculation could be kept minimal. It brought a big improvement in binning and made the following EM algorithm calculation more reliable.

Our metagenomics study of the *Microcystis* colonies showed that various species were associated with the colonies following subculturing. While the dominant organism in the *Microcystis* colonies was *Microcystis wesenbergii* as expected, at least 8 other microbes were found. Analysis of the published metagenomic samples from Lake Taihu showed that these bacteria were present in natural environments (Li et al., 2011; Steffen et al., 2012), suggesting that they were associated with *Microcystis* colonies before the colonies were isolated (Supplementary Table 3). For example, Li et al. (2011) studied the microbial and functional diversity of a bloom in Lake Taihu by Roche 454 platform, eight groups (bacteria species) associated with *Microcystis* in our results were consistent with the taxonomic groups detected by the 454 platform. Our results were also in agreement with the taxonomy of heterotrophic bacteria isolated from *Microcystis* colonies (Shen et al., 2011). Another example is that Group 8 was assigned to *Pseudomonas*, which is a common genus in the phycosphere (Wu et al., 2007; Berg et al., 2008; Li et al., 2011).

A mutually beneficial relationship among the bacteria in the colonies is observed. The energy and carbon flows among the bacteria in the colonies are predictable since *Microcystis* was the only photoautotrophic species in the colonies and the other heterotrophic bacteria depend on *Microcystis* for growth under the culture conditions. On the other hand, *Microcystis* was dependent upon the heterotrophic bacteria for vitamin B-12 that is required for its growth. Analysis of metabolic pathways based on genome information suggested that more than one member of heterotrophic bacteria was involved in biosynthesis of vitamin B12 in the colonies (Figure 4A).

Besides *M. wesenbergii* T100, we have obtained other *Microcystis* from Lake Taihu, including *M. aeruginosa*, and they can be kept forming colonies in the lab. However, when a strain of *M. aeruginosa* was isolated (Yang et al., 2013, 2015) and kept in a bacterium-free condition, it produced much less extracellular polysaccharides and lost the ability to form colony. This observation and the reports that bacteria play a role in colony formation (Shen et al., 2011; Wang et al., 2015) led us speculate that extracellular polysaccharides of *Microcystis* colonies could be synthesized by more than one member of the colony. This view is supported by our analysis of synthesis of lipopolysaccharides (LPS) in the colonies. Among harmful substances produced by *Microcystis* blooms, LPS are classified as endotoxin (Raetz et al., 2007; Wang and Quinn, 2010) and can cause severe diseases in humans and animals (Stewart et al., 2006; Kusumoto et al., 2010). Our metabolic analysis showed that it was unlikely that the *Microcystis* endotoxins were produced by *Microcystis* alone since it didn't contain all the genes required for LPS biosynthesis and one or more members of the *Microcystis*-bacteria community are involved in the process (Best et al., 2003; Bernardová et al., 2008).

Our study demonstrated a mutually beneficial and inter-dependent relationship among the bacteria in *Microcystis* colonies maintained in a subculture condition. It should be realized that the *Microcystis* blooms are more complex and there might be some bacteria that disassociated from the colonies after the long subculture in the lab. It is also worthwhile to note that while our binning method is well suitable for datasets derived from microbial communities with stable species composition (up to 30 species based on the test of published data) (Supplementary Figure S7, Supplementary Table 4), the resolving power of this method could be limited in situations where the species in a community are closely related in phylogeny, or a large number of species is present in a community and co-assembly problem could not be prevented. Nevertheless, the metagenomic approach reported in this study and the results of metabolic pathway complementation could serve as a basis for future study of a more complex interaction between *Microcystis* and the associated bacteria in natural environment.

## Conclusion

Here, we presented a visualization-enhanced binning method and applied it to analyze cyanobacteria-dominated bloom communities from Lake Taihu, China, reconstructing individual bacterial genomes from metagenomic assembly. By analyzing the metabolic pathways of the microbial community, cooperative interactions among the complex species were indicated, which provided insight into the formation mechanism of cyanobacterial blooms.

## Accession codes

The raw sequences data reported in this study have been submitted to the National Center for Biotechnology Information Sequence Read Archive under accession numbers SRX993730 and SRX1007860. This Whole Genome Shotgun project has been deposited at DDBJ/EMBL/GenBank under the accession LFEF00000000. The version described in this paper is version LFEF01000000.

## Author contributions

TL and ZL conceived the study and designed the experiments. ZL, MX, and MR carried out the analysis of data. TL, MX, and MR prepared the first draft of the manuscript. CY contributed materials. HY contributed to the data analysis. ZL, JZ, and TL revised and polished the manuscript. All authors participated in the discussion of manuscript and have agreed to the final content.

### Conflict of interest statement

The authors declare that the research was conducted in the absence of any commercial or financial relationships that could be construed as a potential conflict of interest.
