# Supplementary material for: Metagenomic Analysis Reveals Symbiotic Relationship among Bacteria in Microcystis-Dominated Community
Source: Front Microbiol. 2016 Feb 2;7:56. doi: 10.3389/fmicb.2016.00056 (PMC4735357; doi:10.3389/fmicb.2016.00056)
Supplement: Supplementary file 2 [file DataSheet1.PDF]

Supplementary Material

Metagenomic analysis reveals symbiotic relationship among bacteria in *Microcystis*-dominated community

Meili Xie[1,2,5], Minglei Ren[1,2,5], Chen Yang[1,2], Haisi Yi[1,2], Zhe Li[3*], Tao Li[1*], Jindong Zhao[1,4]

[1]Key Laboratory of Algal Biology, Institute of Hydrobiology, Chinese Academy of Sciences, Wuhan 430072, China
[2]University of Chinese Academy of Sciences, Beijing 100049, China,
[3]State Key Laboratory of Systematic and Evolutionary Botany, Institute of Botany, Chinese Academy of Sciences, Beijing 100093, China
[4]College of Life Science, Peking University, Beijing 100871, China

***Corresponding author:**
Tao Li, Key Laboratory of Algal Biology, Institute of Hydrobiology, Chinese Academy of Sciences, Wuhan 430072, China , E-mail:litao@ihb.ac.cn;
Zhe Li, State Key Laboratory of Systematic and Evolutionary Botany, Institute of Botany, Chinese Academy of Sciences, Beijing 100093, China, E-mail: lizhe@ibcas.ac.cn;

1 Supplementary Figures

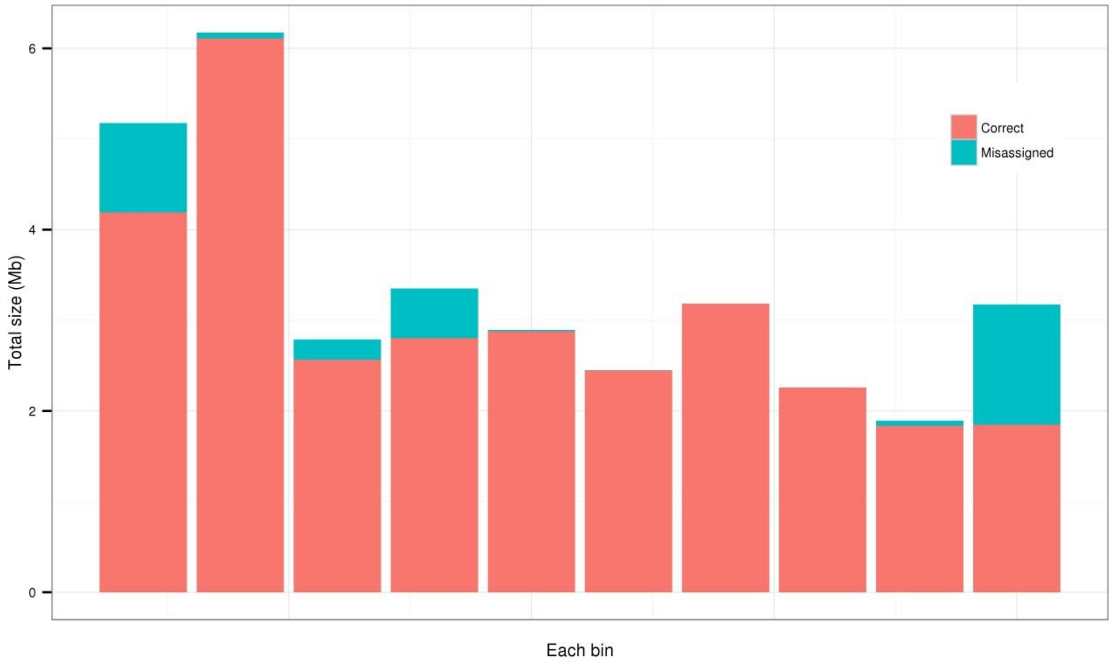

Supplementary Figure S1: Reanalysis of a published simulated metagenomics dataset using our binning method. In the barplot, each bar represents a genome bin, where the majority of sequences are correctly assigned.

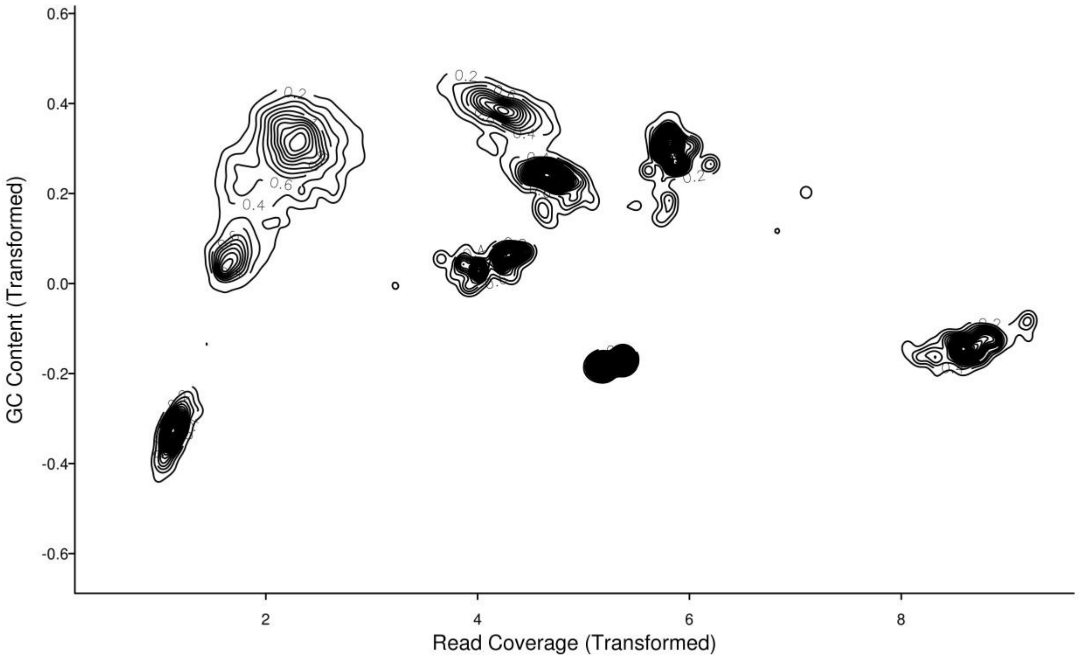

Supplementary Figure S2: Contour plot based on the density of all metagenomic scaffolds at the nucleotide level. The 2-dimentional kernel density estimation was used to explore the underlying probability density function of GC content and coverage information of each scaffold. Before the calculation of density, each point was weighted by the length of corresponding scaffold. Based on the result, the contour is created to display an overall picture of structure of the dataset.

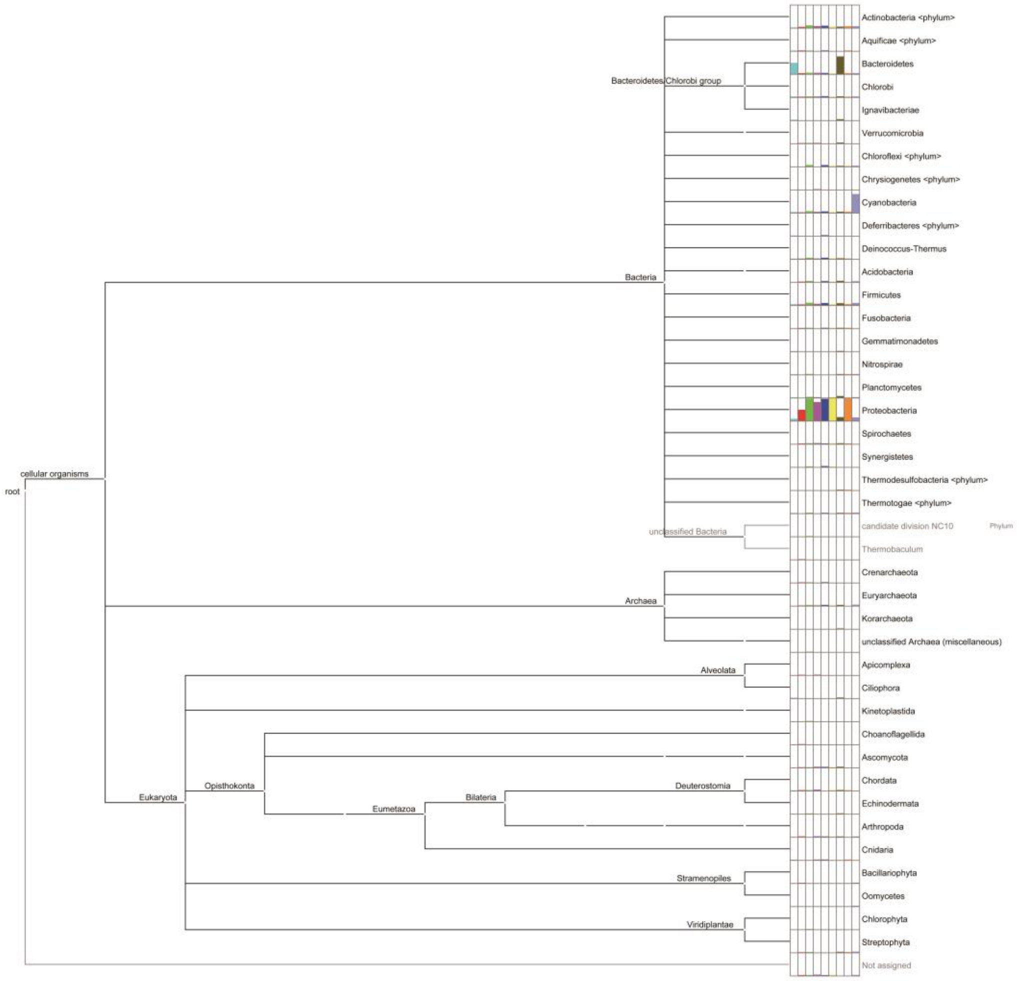

Supplementary Figure S3: Taxonomic assignment of the microbial community at the phylum level. Each color represents a group, and the height of the column displays the quantity of the gene in the corresponding group. The figure was generated by the metagenomics analysis tool MEGAN.

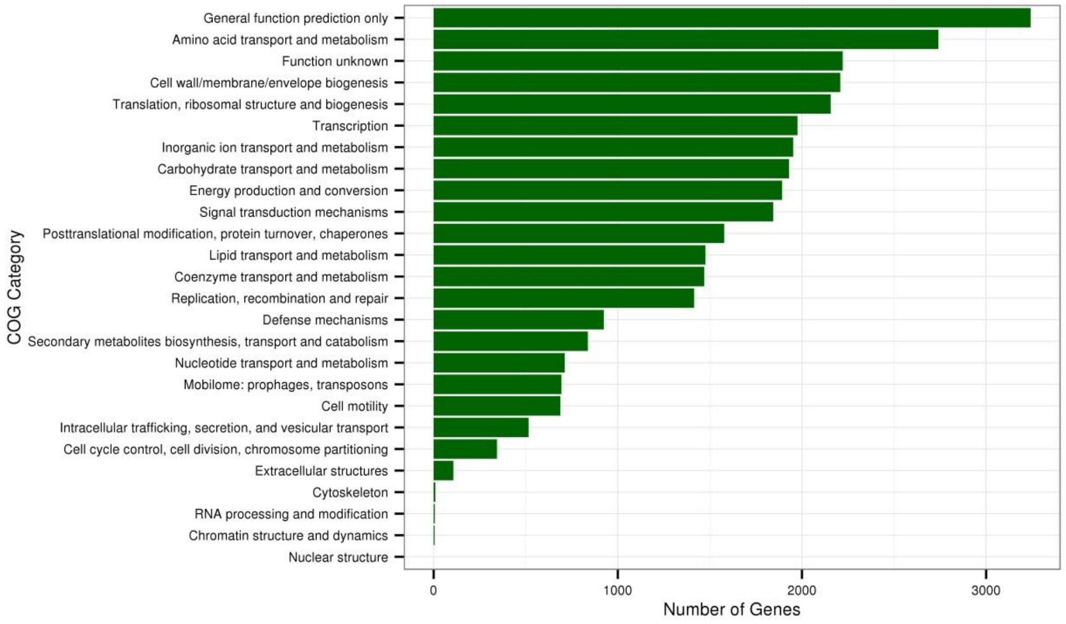

(A)

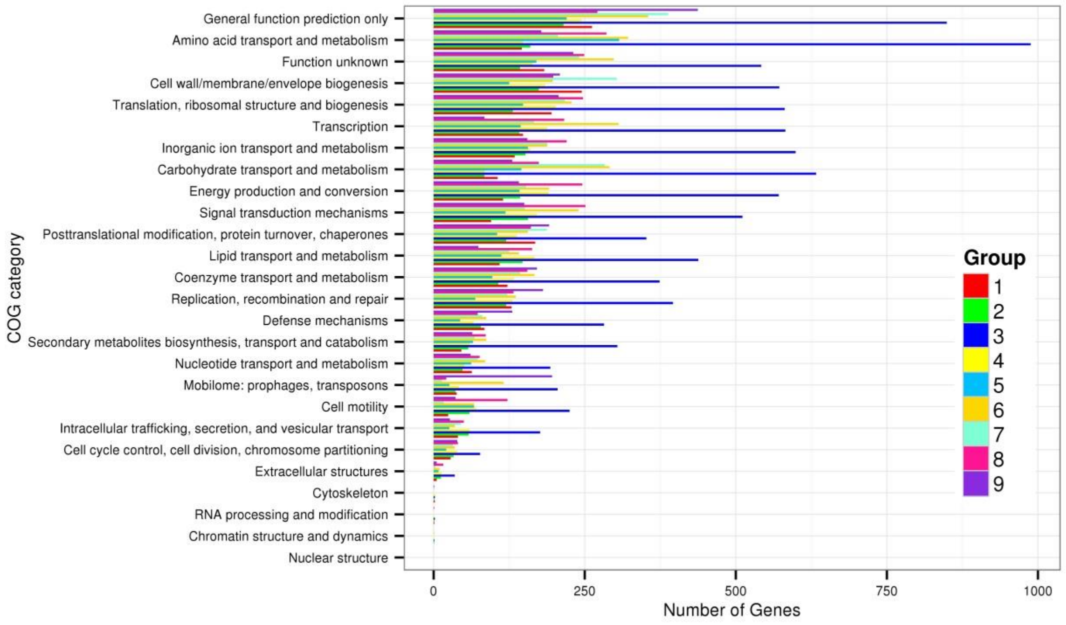

(B)

Supplementary Figure S4: The numbers of predicted genes assigned to each COG category. The microbial communities were annotated using the COG database. (A): The distribution of genes of the microbial community in each COG category. (B): The distribution of genes of each group in each COG category.

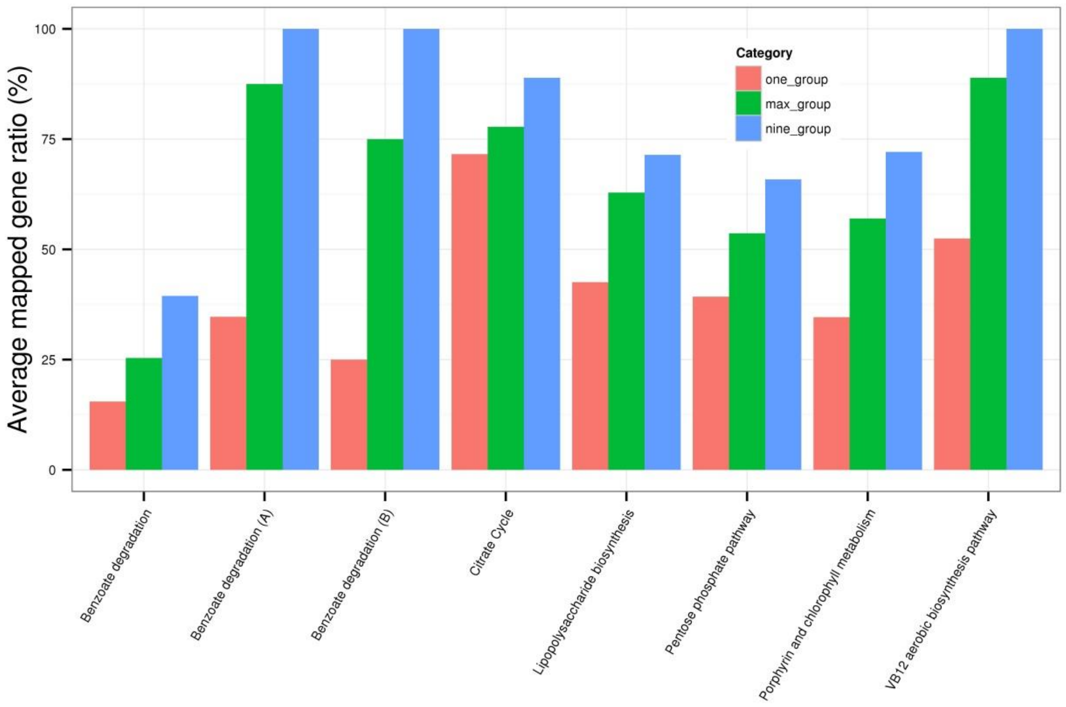

Supplementary Figure S5: A complementation ratio of the metabolic pathways. The blue bars are the average mapped gene proportions of nine groups, the red bars are the combined nine groups, and the green bars are the maximum ratio among nine group. Note the ratio is the proportion of the mapped genes in all the genes required in each stand-alone pathway, which is used in Endo *et al* (see the manuscript).

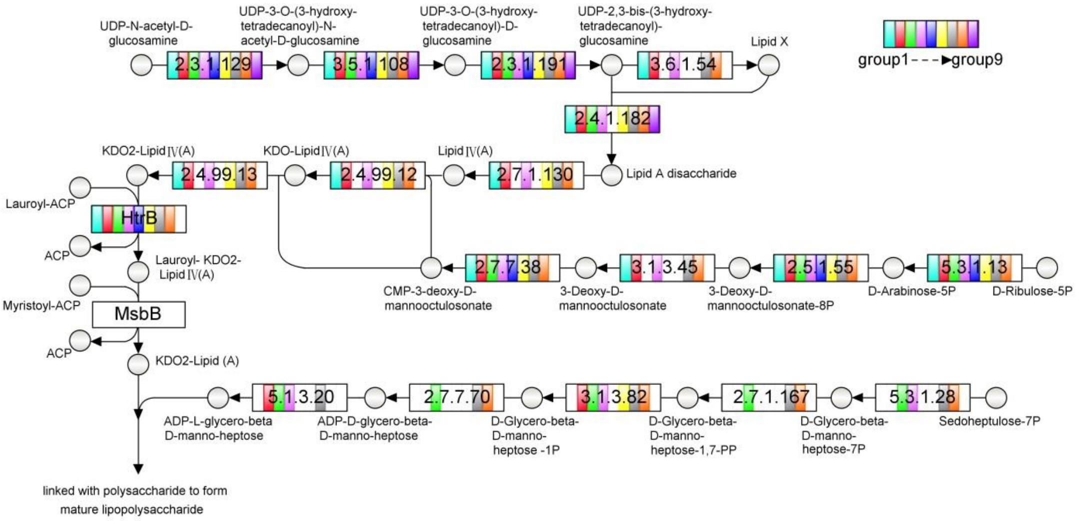

Supplementary Figure S6: The metabolic pathway of Lipopolysaccharide biosynthesis. The circles represent the intermediates, the rectangles represent the enzymes, while each color represents a group, and the white color indicates the absence of the corresponding enzyme in this group.

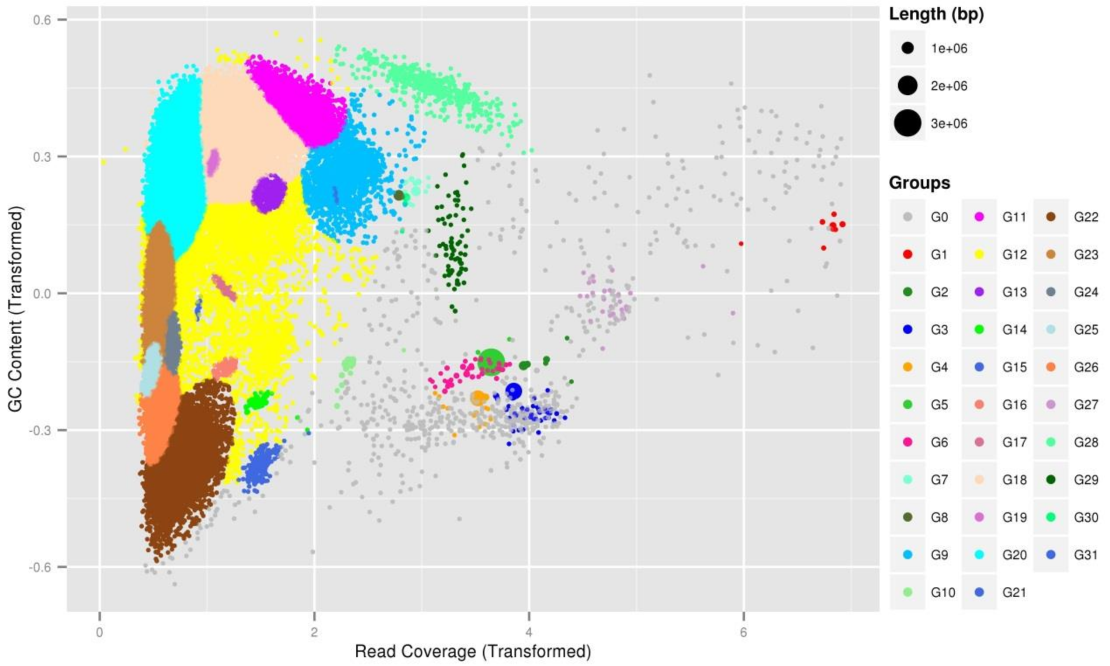

Supplementary Figure S7: Scatterplot of the final binning results based on the reanalysis of the metagenomics sample from Albertson's article. The meaning of all symbols is the same as the Figure 2.

2 Supplementary Tables

Supplementary Table 1

The contigs list of each group of *Microcystis* colonies

See Supplementary_Table1.xlsx (in Excel)

Supplementary Table 2

Statistics of the essential genes in each group of *Microcystis* colonies

Group	total_ess	unique_ess	duplicated_ess
Group 1	102	100	1
Group 2	48	46	2
Group 3	275	91	70
Group 4	112	106	4
Group 5	94	93	1
Group 6	108	104	4
Group 7	106	105	1
Group 8	111	105	7
Group 9	103	95	5

Supplementary Table 3

Comparison with the published datasets (both about cyanobacterial bloom in Lake Taihu)

Group	the datasets in Li et al (2011)		the datasets in Steffen et al (2012)	
	no_mapped_reads	relative abundance(%)	no_mapped_reads	relative abundance(%)
Group1	115	0.02	1,565	0.39
Group2	995	0.19	1,067	0.27
Group3	20,426	3.96	17,537	4.36
Group4	988	0.19	1,277	0.32
Group5	9,300	1.8	9,418	2.34
Group6	10,907	2.12	7,126	1.77
Group7	598	0.12	361	0.09
Group8	3,686	0.72	4,450	1.11
Group9	180,166	32.9	64,175	15.96

Supplementary Table 4

The basic information for each genome bin of the published dataset

group_name	total_ess	unique_ess	duplicated_ess	no_scaffold	total_base	gc_content
group_C0	143	78	48	3518	19,022,108	0.5
group_C1	57	53	4	9	1,012,627	0.57
group_C2	52	52	0	6	1,343,859	0.42
group_C3	129	91	37	43	3,127,961	0.39
group_C4	71	61	8	16	2,486,895	0.39
group_C5	65	60	5	4	3,357,390	0.42
group_C6	65	64	1	39	2,373,029	0.42
group_C7	21	21	0	4	1,282,175	0.61
group_C8	42	41	1	3	1,265,234	0.61
group_C9	239	105	78	1121	9,892,646	0.62
group_C10	120	104	16	18	3,663,960	0.42
group_C11	192	93	56	2062	8,379,303	0.61
group_C12	98	66	23	995	6,010,784	0.62
group_C13	163	101	54	554	9,551,521	0.6
group_C14	114	104	10	45	4,311,419	0.38
group_C15	69	65	4	184	3,097,591	0.33
group_C16	84	68	15	137	2,322,542	0.43
group_C17	227	106	82	135	4,904,213	0.51
group_C18	395	107	99	4904	20,592,880	0.59
group_C19	154	88	47	1636	9,136,461	0.6
group_C20	1196	107	106	20984	61,728,555	0.57
group_C21	110	97	12	35	1,463,090	0.49
group_C22	280	94	71	4858	25,457,199	0.44
group_C23	237	94	70	5598	15,104,482	0.55
group_C24	141	92	39	804	8,054,384	0.47
group_C25	80	60	18	1693	4,521,048	0.55
group_C26	261	103	75	4387	13,217,844	0.52
group_C27	84	82	1	32	592,082	0.49
group_C28	84	66	18	405	2,278,607	0.66
group_C29	89	88	1	32	846,113	0.53
group_C30	50	46	4	5	649,732	0.6
group_C31	104	72	32	99	1,025,865	0.6

170
